# The Development and Testing of a Relationship Skills Intervention to Improve HIV Prevention Uptake Among Young Gay, Bisexual, and Other Men Who Have Sex With Men and Their Primary Partners (We Prevent): Protocol for a Randomized Controlled Trial

**DOI:** 10.2196/10370

**Published:** 2019-01-02

**Authors:** Kristi E Gamarel, Lynae A Darbes, Lisa Hightow-Weidman, Patrick Sullivan, Rob Stephenson

**Affiliations:** 1 Department of Health Behavior and Health Education School of Public Health University of Michigan Ann Arbor, MI United States; 2 Center for Sexuality and Health Disparities University of Michigan Ann Arbor, MI United States; 3 Department of Health Behavior and Biological Sciences School of Nursing University of Michigan Ann Arbor, MI United States; 4 Institute for Global Health and Infectious Diseases The University of North Carolina at Chapel Hill Chapel Hill, NC United States; 5 Department of Epidemiology Rollins School of Public Health Emory University Atlanta, GA United States

**Keywords:** HIV, telehealth, men who have sex with men, adolescents, couples

## Abstract

**Background:**

Young gay, bisexual, and other men who have sex with men (YMSM) continue to be the group most heavily impacted by HIV in the United States. Substantial evidence indicates that up to two-thirds of new HIV infections occur in the context of a main partnership. Couples HIV testing and counseling (CHTC) has been shown to be a promising and effective strategy for increasing HIV prevention uptake among male couples; however, YMSM who are new to relationships may not have yet developed the efficacy, negotiation, and communication skills to navigate HIV testing in their relationship and communicate around developing a prevention plan.

**Objective:**

This study aims to develop and test a relationship skills–focused HIV prevention intervention for YMSM and their partners. The intervention consists of two telehealth-delivered sessions: the first focuses on relationship skills and the second consists of CHTC and prevention planning. Both sessions are attended by both members of the dyad.

**Methods:**

This protocol describes the development of the proposed intervention (*We Prevent*) and pilot test to examine its feasibility and preliminary efficacy. The intervention will include two motivational interviewing–based sessions: session one is a relationship skills–building session, focused on techniques to explore and build communication skills in a relationship, to help YMSM develop and enhance necessary skills for their current and future relationships; the second session is a CHTC session with YMSM and their partners, to help them develop an HIV prevention plan. Through qualitative data collection and a one-arm pilot with YMSM, we will develop and refine a developmentally appropriate relationship skills session as an addition to the current CHTC intervention. We will then conduct a pilot randomized controlled trial (RCT), comparing the acceptability, feasibility, and preliminary efficacy of the adapted two-session telehealth intervention for YMSM versus a control group receiving one session only—a CHTC session delivered via telehealth.

**Results:**

The *We Prevent* intervention is designed to increase uptake of HIV prevention, shown through self-reported reductions in condomless sex and increases in knowledge and uptake of pre-exposure prophylaxis. In addition, the intervention is designed to increase HIV and sexually transmitted infection (STI) testing. STI incidence is examined as a secondary outcome. A cost-input analysis will examine the costs associated with intervention delivery to inform future scale-up of the intervention.

**Conclusions:**

Drawing on theory and existing CHTC protocols delivered with video-based counseling, this proposed intervention affords the opportunity to empower YMSM with the skills necessary to communicate with their partners and protect themselves from HIV in their current and future relationships.

**Trial Registration:**

Clinicaltrials.gov NCT03551938; https://clinicaltrials.gov/ct2/show/NCT03551938 (Archived by WebCite at http://www.webcitation.org/73omJCz1a)

**International Registered Report Identifier (IRRID):**

RR1-10.2196/10370

## Introduction

### Background

Young gay, bisexual, and other men who have sex with men (YMSM) continue to be the group most heavily affected by HIV/AIDS in the United States [1,2], with increasing incidence of HIV identified among YMSM of color, specifically African-American and Latino youth [1]. Approximately 14% of youth living with HIV are estimated to be unaware of their serostatus, of whom 52% are those assigned a male sex at birth and have sex with men [1].

A number of individual and social factors (eg, substance use, mental health, poverty, stigma, incarceration, and homelessness) have been associated with increased HIV incidence among YMSM [3]. Notably, epidemiological evidence illustrates that up to 80% of new HIV infections occur from primary partners among YMSM [4,5], highlighting the urgent need to attend to the relationship context of HIV transmission in this population [6].

Numerous studies suggest that individuals are more likely to have condomless anal sex (CAS) with their primary partner as compared with casual partners [4,7], and relationship factors such as intimacy, closeness, and trust have been identified as powerful motivators for CAS in relationships [8-10]. For many individuals, there might be an underlying belief that condoms are antithetical to intimacy and that having condomless sex with their partners connotes an act of intimacy [11,12]. However, increased HIV transmission risk among YMSM may occur when a partner lacks knowledge of their own or their partner’s serostatus before engaging in CAS [13-18]. For example, studies have estimated that more than 80% of HIV-negative YMSM practice CAS within their relationships [14,18]. Furthermore, some of these young men also engage in CAS outside of their relationship (ie, concurrent CAS). Engagement in CAS (monogamous or concurrent) combined with low rates of testing for HIV and other sexually transmitted infections (STIs), without confirming one’s own or a partner’s HIV serostatus as negative, heightens YMSM’s vulnerability to HIV and other STIs [14,15,18,19].

Despite mounting evidence that dyadic approaches are generally efficacious in promoting safer sex behaviors in adult populations [20-22], few dyadic HIV prevention interventions exist for YMSM in relationships [20,23]. To date, couples HIV testing and counseling (CHTC) represents one of the very few effective couples-focused HIV prevention interventions for male couples. CHTC has been used as an HIV prevention for heterosexual couples in Africa for over 20 years [24] and was adapted for male couples in the United States [25-27]. CHTC has been shown to be effective for male couples in promoting the formation and adherence to prevention planning. It is now endorsed by the Centers for Disease Control and Prevention (CDC) as an effective HIV prevention strategy and is being used across the United States [25,28]. In contrast to individual HIV testing and counseling, CHTC includes both partners in 1 session where they receive counseling and testing together at the same time. Specifically, in the single 30- to 60-min CHTC session, the counselor learns about the couples’ relationship and provides tailored counseling and prevention recommendations based on the relationship and serostatus results [24,26].

Although CHTC holds promise in reducing HIV incidence among male couples in general, young male couples may lack the behavioral skills necessary to undergo HIV testing with their partner. YMSM report infrequent HIV testing, even when CAS has occurred with outside partners [29,30]. With few exceptions [31], the majority HIV prevention strategies aimed at YMSM have focused in large part on reducing condomless sex with casual partners [32], effectively ignoring the role of relationships in shaping HIV risk. Thus, YMSM may not perceive themselves to be at risk of HIV acquisition from their primary partner and may lack skills, such as assertive and effective communication, around negotiating relationship dynamics [33]. These factors may limit their uptake of CHTC.

HIV prevention interventions are beginning to recognize the role of relationship factors in shaping HIV risk, although they remain largely focused on adult men who have sex with men (MSM). One example is the 2GETHER intervention that was developed and pilot tested with same-sex male couples aged 18 to 29 years [31]. The 2GETHER intervention involved in-person group format and in-person couples sessions, providing relationship and HIV prevention education to adult male couples in an effort to increase knowledge, motivation, and behavioral skills among male dyads. The 2GETHER intervention demonstrated preliminary efficacy in reducing sexual risk behaviors [31]. 2GETHER focused on building behavioral skills within relationships among 18 to 29-year-old male dyads and, although perhaps still considered young adults, 18- to 29-year-olds have more relationship and life experiences than younger adolescents. Youth, aged 15 to 19 years, the target for the *We Prevent* intervention, are often just beginning to explore relationships; they thus have a very different set of informational, behavioral, and developmental needs. 2GETHER did not consider the unique developmental needs of MSM below the age of 18 years. *We Prevent* aims to fill this intervention gap through an intervention tailored to the specific needs of 15- to 19-year-old male dyads. By providing a web-based intervention, unlike the in-person format of 2GETHER, *We Prevent* also aims to allow young male couples to receive interventions in an environment in which they feel comfortable, surmounting structural barriers to accessing services.

Accumulating evidence also supports the efficacy of motivationally focused behavioral interventions to reduce HIV transmission risk among YMSM who may not perceive themselves to be at risk for HIV [34,35]. Therefore, motivational interviewing (MI) techniques may be particularly helpful alongside relationship and HIV prevention education to help YMSM develop the skills necessary to navigate the complexities of HIV prevention in their romantic relationships. In addition, the reach of in-person interventions may be restricted by general barriers to dissemination and implementation (eg, cost and highly skilled counselors) [36] as well as the social context of YMSM (eg, fears of being *outed* in their immediate geographical locale) [35,37]. Given the promise of brief technology-delivered interventions (eg, Keep It Up!) [38] and the transitory nature of relationships at this age, younger MSM may benefit from brief online interventions designed to address their relationship and HIV prevention needs.

The proposed intervention, *We Prevent,* will be delivered to young male dyads. The intervention itself, however, is conceptualized as an individual intervention, whereby the aim is to examine how the experience of testing with a male partner, along with relationships-specific skills building, can promote the development of relationship skills that have a long-term influence on the individual’s relationships and, subsequently, their engagement in HIV prevention and care. That is, the intervention is intended to create behavioral change at the individual level; although participants receive the intervention as a dyad, it is expected that many relationships in this age group are relatively short in length. The *We Prevent* intervention thus aims to equip individuals with relationship skills to use in their current and future relationships.

As described above, YMSM often rely on online technologies to build their social and sexual networks, receive social support, and obtain relevant health information. In general, internet use among youth and young adults aged 15 to 29 years is nearly universal, at 99% in 2016 [37,39]. Thus, telehealth offers the opportunity to disseminate HIV prevention strategies to YMSM who might otherwise not have this opportunity. Telehealth aims to circumvent traditional impediments to health care access. Over the past decade, telehealth formats have been adapted for use in MSM populations where stigma and a lack of lesbian, gay, bisexual, transgender, queer (LGBTQ)–friendly health care providers contribute to reduced access to care [40]. Online interventions are seen as convenient for youth users and allow for home-based access to health messaging, thereby reducing fears of embarrassment or *outing* by connecting with local resources [36,41]. Recently, CHTC was adapted and is currently being tested using telehealth, specifically videoconferencing software, with preliminary evidence showing high feasibility and acceptability [26]. Both sessions of the *We Prevent* intervention will be delivered virtually via a Health Insurance Portability and Accountability Act (HIPAA)–compliant remote video chat service.

### Theoretical Framework For We Prevent

*We Prevent* draws on the Relationship-Oriented Information- Motivation-Behavioral Skills (RELO-IMB) model [42], which is premised on the IMB model [43]. RELO-IMB was developed for YMSM communities [31]. The *Information* component of the RELO-IMB model is addressed by targeting YMSM-specific knowledge (eg, risk within dyads and with outside partners), *Motivation* is targeted by addressing attitudes and peer norms about HIV prevention in relationships, and *Behavioral Skills* is targeted by addressing risk-reduction skills relevant to YMSM and their partners (eg, discussion about safer sex, HIV testing, and negotiating safety in one’s sexual agreement).

*We Prevent* uses MI techniques and includes 2 sessions: session one—a motivational interview-guided session that provides a facilitated discussion between YMSM and their partner, in which they explore and come to understand their own HIV risk and learn behavioral skills to improve communication—and session two—a CHTC session between the same YMSM and their partner, which facilitates the development of a prevention plan that meets the goals of both partners. In contrast to existing couples-based HIV prevention interventions, *We Prevent* is conceptualized as an individual intervention, whereby the aim is to promote development of relationship skills that can have a long-term influence on the individual’s relationships and, subsequently, their engagement in HIV prevention and care.

Accordingly, we hypothesize that YMSM and their partners who engage in *We Prevent* will demonstrate reductions in sexual risk (eg, STI incidence) and greater knowledge and awareness of different prevention options (eg, knowledge of pre-exposure prophylaxis [PrEP] and the importance of repeat STI and HIV testing). We also hypothesize that YMSM who engage in *We Prevent* will demonstrate greater communication skills to use in their relationship, which will provide them with greater self-efficacy for developing an HIV prevention plan with their partner.

### Aims and Objectives

The aim of this paper was to describe the protocol for the refinement, pilot testing, and pilot randomized controlled trial (RCT) to examine the acceptability and feasibility of the *We Prevent* intervention.

*We Prevent* will work closely with the innovative technology (iTech) cores on the development, testing, and analysis elements of each of the 3 phases of research activities described below. Although recruitment is not restricted to the iTech subject recruitment venues (SRVs), the iTech technology core (TC) will provide guidance on the technology necessary for recruitment and retention strategies as well as technology-related ethical issues for conducting the project with YMSM and their partners. The iTech analytic core (AC) will provide oversight for qualitative and quantitative analyses, including cost affordability analyses. The iTech management core will provide assistance with recruitment and regulatory compliance.

## Methods

### Trial Registration, Ethics, Consent, and Institutional Board Approval

This study has been reviewed and approved by the University of North Carolina Institutional Review Board (IRB# 18-0200). Reliance agreements were established for each SRV. A certificate of confidentiality has been obtained from the National Institute of Child Health and Human Development, and a waiver of parental consent/assent will be obtained for participants aged 15 to 17 years. The study will also be registered on ClinicalTrials.gov.

### Overview of Study Design

The study includes 3 phases. Phase I will collect qualitative data from YMSM and feedback from technical experts to develop and refine the 2 sessions the intervention comprises. Phase II involves a one-arm pilot of *We Prevent*, where we will examine the feasibility and acceptability of the intervention as well as its impact on self-reported HIV and STI testing and PrEP knowledge and uptake. We will also analyze the intervention’s impact on STI and condomless sex incidence. Phase II will enroll 20 YMSM couples (40 individuals) using online recruitment strategies. Phase III involves a pilot RCT of *We Prevent* (experimental condition) compared with the existing CHTC intervention only (control condition). The pilot RCT will recruit 160 YMSM and their partners (ie, 320 individuals), randomized 1:1 to the intervention and control groups. Primary outcomes of the pilot RCT include uptake of HIV prevention, defined as self-reported routine HIV and STI testing, increased PrEP knowledge and use, and reductions in condomless sex. STI incidence is examined as a secondary outcome, with biomarkers of STIs collected through self-samples that are mailed in by participants. Data on costs associated with the delivery of the intervention are collected to allow a cost analysis to inform the future scale-up of the intervention.

### Participants

For all phases, participants must be (1) between the ages of 15 and 19 years; however, the age of recruitment will vary by state because of variations in state consent law such that in some states we will not be able to recruit participants who are as young as 15 years; (2) identify that they are in an emotional and/or sexual relationship with another male (assessed through multiple questions); (3) born male and identify as male; (4) report that they engaged in CAS in their lifetime; (5) are willing to have HIV and STI testing kits delivered to an address that they provide (for phases II and III); (6) have access to a computer with internet access within their home (or the home of one partner); and (7) self-report being HIV negative or unknown serostatus. Male partners must meet the same criteria with the exception of the age restriction and HIV status. We will exclude those who report a recent (within the past 6 months) history of any intimate partner violence, using methods specifically designed for use with male couples, which involves continuously monitoring the presence of intimate partner violence at each assessment point [44]. Partners’ age criteria will vary by state laws regarding age of consent laws and statutory rape laws such that we will not be able to recruit some partners who are as young as 15 years in some states (eg, California) or 2 years older than a participant in other states (eg, Colorado).

### Recruitment

For all phases, participants will be recruited from across the United States via online advertisements placed on key social media websites (eg, Facebook) and social media sites aimed specifically at MSM (eg, Grindr). Working with iTech TC, the online ads will show visual representations of young male couples in a range of races/ethnicities and will be titled *We Prevent*.

YMSM who click on the advertisement will be taken to a Web page that provides basic information on the study. YMSM proceed to an assent Web page and provide an electronic assent/consent. After assent/consent, YMSM will complete a short screener to assess eligibility and will provide their and their partner’s contact information. YMSM who do not provide assent/consent, meet the eligibility criteria, or provide complete contact information will be excluded from the study and be redirected to the online CDC HIV toolkit.

Eligible YMSM will continue by registering. During the registration process, they will provide their contact information and a nickname or name of choice. YMSM who register will be provided with a link via email to allow them to continue to set up an account by selecting a username, password, and security questions. Once the index case (the YMSM who initially clicks on the advertisement) and their partner have both completed the assent forms and the screening questionnaire, both partners have proven eligible for the study, and both have registered on the study website, emails will be sent to the participants detailing the information for their next steps of project participation. For phases II and III, couples’ eligibility will be verified post hoc by assessing concordance in both partners’ responses to items in the eligibility screener. These post hoc verification procedures have been successfully used in other studies with male couples [45]. Study staff will follow up with a phone call to go over study logistics and will confirm the contact information for each participant. We have been granted a waiver of parental consent for youth aged 15 to 17 years. During the call, the study staff member will review the consent process to ensure they understood their rights and the details of their participation. For phases II and III of the study, participants will be asked to provide a mailing address to receive HIV testing kits (for the second intervention session) and STI testing kits (for the assessment of STI incidence as a secondary outcome). Participants are informed that they can choose any address other than a post office box. In a recent feasibility of home-based HIV testing and remote video counseling with transgender youth aged 15 to 24 years, 100% of the 201 participants provided a delivery address for HIV testing kits, 100% received the kits, and 98% reported their HIV test results [46]. Tests kits are delivered in unmarked boxes, and participants are informed of their delivery date. We will use similar procedures to ensure the safety of participants in phases II and III of the activities.

### Phase I Study Procedures

The first phase involves in-depth interviews (IDIs) with YMSM, technical expert group (TEG) reviews, and theater testing and cognitive review of the intervention. These data will be used to revise the content and study procedures in preparation for implementation.

#### In-depth Interviews With Young Men Who Have Sex With Men

For this task, 40 YMSM will be recruited to participate in IDIs using the recruitment methodology and eligibility criteria outlined above. Partners of index participants will not be recruited or engage in phase I activities. The 40 YMSM will be stratified by race: 10 white, 15 Latino, and 15 black. The IDI will be conducted via VSee, a HIPAA secure video chat software that will also be used to deliver the intervention and that has been used in prior studies [26].

One way that participants can actively guide a qualitative interview process is through the use of activities in which participants are given guidelines or instructions by the researcher but then take control of the activity in a flexible and participant-centered approach. Qualitative data collection involving visualizations can be useful to convey depth and detail that expand beyond verbal expression [47].

The IDI will adopt such a participatory methods approach. During the IDI, participants will create a visual relationship timeline using virtual stickers to develop an overview of their dating and sexual history. The IDI follows a step-by-step process where participants place stickers on the timeline in response to questions about relationship dynamics, desires, and communication. To construct the timeline, participants will add nonidentifying nicknames for up to 5 “sexual and/or romantic partners” who were “significant or memorable” to the participant in some way; participants will define for themselves what “significant or memorable” means.

The timeline begins when the participant first met the earliest partner and ends at the present time. Lines are added to show when and how long each relationship occurred. Participants are given flexibility on how to draw the lines to best represent the timeline of their relationship history (eg, participants could choose to use different types of lines to represent different parts of the relationship, lines could stop and start again, and lines for different people could overlap over the same time period).

Participants then answer a series of questions on each relationship through an action-oriented process that involves applying stickers with predetermined labels to the timelines. Participants will first use “relationship tag” stickers with definition terms (eg, partner, boyfriend, and friends with benefits). Follow-up questions examine why terms were chosen, definitions of terms, relationship development and transitions, and relationship rules (eg, monogamous vs open relationship). Participants then answer the question, “How did you feel about this person when you were together?” by adding up to 5 positive and/or negative “emotion tags” for each partner (eg, trusting, loved, disrespected, and not myself).

The timeline provides an anchor for discussion around relationship communication, negotiation, and desires. Using the timeline, participants will be asked to define what a relationship is, their definition of a successful relationship, and their desires for future relationships. Participants will be asked to describe positive and negative experiences they have had in communicating within relationships. The IDI will ask participants to outline the communication skills they believe they have and the communication skills they desire to have. It will end by asking the participants to describe their desired content and quality for a relationship skills–focused facilitated session. The goals and suggested outline of the session will be described, and participants will be asked to make suggestions for specific content areas.

#### Adaptation and Technical Expert Review

On the basis of findings from the IDI, the content of the relationship skills session will be developed, and the adaptation of the existing CHTC session will occur. The adaptation of the intervention is to ensure that the content is developmentally appropriate (eg, language and content) and the relationship skills building session meets the needs of YMSM aged 15 to 19 years. A TEG will be formed, consisting of members who engage with diverse communities of YMSM and have experience in the provision of HIV and LGBTQ clinical and social services.

After modifying the intervention content, a series of meetings will be scheduled individually with the TEG members to (1) review the intervention content and training protocols for the 2 counseling sessions and (2) explore existing screening and assessment tools that are culturally and linguistically appropriate for use with diverse groups of YMSM and their partners. VSee video chat will be used for TEG discussions focused on the adaptation of intervention assessments and content as well as the development or provision of feedback associated with the counseling components of the intervention.

In addition, a youth advisory board (YAB) of approximately 8 YMSM who meet the same eligibility criteria as for research participants will be convened. The YAB will be involved in providing feedback on the adaptation of the intervention and study protocols. They will be asked to meet the investigative team 2 to 3 times during the adaptation phase as well as provide feedback on different aspects of the project, including feedback on logo and recruitment materials, website design, website content, and intervention language.

#### Theatre Testing and Cognitive Review

To develop, refine, and standardize the intervention’s content, we will use best practices in usability testing to examine the preliminary intervention. Individual usability interviews with YMSM (n=10) will be conducted using the same recruitment procedures outlined in the IDI stage above. During usability interviews, the moderator will walk the participant through each portion of the intervention manual. Similar to cognitive interviews, they will be asked to think aloud as they navigate through the intervention. The moderator will note the participant’s behavior and any questions that they have regarding the content and flow. As they navigate through the intervention, recordings will be made of any nonverbal behavior that could be important to take into consideration (eg, frowns, sighs, or fidgeting). Recordings will be made of valuable data related to how they respond to each module (eg, how long does it take participants to understand and respond to different modules?). These data will be used as exploratory indicators of content difficulty, attentiveness, and task difficulty. After participants have completed the assessment, they will be asked to reflect on whether the intervention met or exceeded their expectations and their HIV prevention and relationship needs. These data will be used to revise content and study procedures in preparation for implementation.

### Phase I Data Analyses

All video interviews will be audio-recorded and transcribed verbatim. With guidance from the iTech AC, we will use framework analyses for all qualitative analyses [48]. Framework analyses are systematic and dynamic in their approach to qualitative data, resulting in the ability to produce accessible analyses focused on specific research questions. The thematic framework will be refined for coding by reading and rereading the data, identifying themes that emerge, and writing analytical memos about those themes. Next, specific sections will be identified that corresponded to particular themes. Finally, we will refine the relationship between indexed data and the original thematic framework, interpreting the resulting themes. Reliability among the coders will be checked by having each coder code a subset of transcripts, with acceptable agreement defined as ≥90% reliability. Disagreements will be resolved through discussion with a third party. Qualitative analyses will involve identifying and summarizing patterns of experiences related to the intervention manual and identifying how to improve the intervention. The study team will review the analysis of qualitative data and assess the strengths and weaknesses of each of the components of the intervention manual based on the findings. The research team will meet TEG and YAB to share results and discuss how best to improve the intervention modules, exercises, and process.

### Phase II Study Procedures

We will conduct a one-arm pilot of *We Prevent* to examine the acceptability and feasibility of the intervention and examine the impact of the intervention on increasing self-reported HIV and STI testing and PrEP knowledge and use. We will also examine the feasibility and acceptability of home STI collection kits for laboratory-confirmed STI incidence. These study findings will be used to inform any necessary modifications to phase III RCT. Phase II will be a prospective study, enrolling a sample of 20 YMSM couples (40 individuals). Recruitment and eligibility screening will mirror the procedures for phase I. As described above, couples’ eligibility to enroll in the study will be verified post hoc by assessing both partners’ responses to items in the eligibility screener for phases II and III. After the completion of the second intervention session, both participants will complete an immediate self-administered follow-up survey and qualitative exit interview as well as 3-month follow-up surveys and STI home tests.

#### Recruitment, Registration, and Retention

After registering, assented participants will complete a 25-min baseline questionnaire. For interventions to be evaluated as potential *best evidence* –based interventions through CDC’s Prevention Synthesis Research activity, data must be available for at least a single follow-up time point for at least 70% of participants. We will use best practices to retain participants (eg, comprehensive locator information that includes participants’ cell phone numbers and email), while being sensitive to the risk of undue disclosure of YMSM participating in the study. A certificate of confidentiality will be obtained from the National Institute of Child Health and Human Development, and a waiver of parental consent/assent will be obtained for participants aged 15 to 17 years. In addition, we allow participants to specify the day of the week and time of day when they would like to receive electronic follow-up surveys. Depending on the participant’s preferences provided upon registration, contacts will be made initially with the preferred mode of recontact (eg, by short message service text message); if still unresponsive, other available modes (eg, phone call) will be used.

Recruitment and retention activities will be monitored through a participant management system that maintains electronic lists of participants’ retention status and automatically creates notification lists for retention staff to ensure that a systematic process is followed and carefully documented for retention. We will follow YMSM for 3 months. The short time frames between assessments help us to respond quickly to retention concerns. Incentives for completing the baseline and follow-ups will be US $40 per assessment.

Index participants and their partners will first complete the baseline survey and then be taken to an online calendar asking them to schedule their first intervention session. The calendar will be populated by study staff per their availability and will reflect local time zones. The page will explain the 2-session intervention format, will provide detailed instructions on downloading the VSee video chat software, and will contain a list of instructions for receiving the intervention (ie, both partners must be together, audio and visual privacy). The VSee software can be used on a personal computer, tablet, or any mobile platform [26]. After the first session, the index participant and their partner will be mailed an HIV testing kit to be used in their second session.

#### Intervention Condition

The intervention consists of 2 45-min sessions, timed approximately 2 weeks apart. The first session will focus on defining healthy and unhealthy characteristics of relationships, teaching and practicing effective communication skills, reviewing couples-based sexual health information (ie, negotiated safety, PrEP, and HIV and STI testing), and preparing for engaging in CHTC as a couple. The second session—the CHTC session—will follow a similar format to the existing CHTC counseling protocols, the same format as provided to couples in the control condition (ie, pretest counseling, HIV testing, discussion of HIV risks, delivery of test results, and posttest counseling). Specifically, both *We Prevent* sessions are designed to help YMSM and their partners learn and practice communication skills and set goals regarding HIV prevention and care that can be used throughout their lives.

Participants who receive an HIV-positive result will be counseled on the need for timely linkage to care. The counselor will arrange a time within 1 week of the initial session to conduct a second video session for couples in which one or both of them have preliminary positive results. During this session, new preliminary positives will be directly linked to medical care in their local area. Study staff will follow up with them on the next business day to ensure that contact was made with a local facility closest to where the participant lives or with a medical care agency. The participant would be contacted at least three times to (1) confirm an appointment was scheduled, (2) confirm the appointment was attended, and (3) report confirmatory results.

#### Team Review and Data Analyses

All sessions and qualitative exit interviews will be audiotaped (with participant consent). Audiotapes will be reviewed by the investigative team, TEG, and members of the iTech AC. This team will conduct a conference call every other week to assess the strengths and weaknesses of the intervention components and indicate revisions to the preliminary protocol. At the completion of intervention for each dyad, the review will focus on potential changes to the protocol (eg, content and timing of interventions and sessions) that will be implemented before the next set of participants starting the intervention. This process will lead to a finalized version of the *We Prevent* manual. We will then examine the impact of the *We Prevent* intervention condition on feasibility, acceptability, and preliminary promise in reducing HIV risk (see phase III for a detailed description of measures, benchmarks, and analyses).

### Phase III Study Procedures

We will conduct a pilot two-arm prospective RCT to compare the preliminary efficacy of *We Prevent* versus CHTC alone, both delivered through video counseling, on increasing self-reported HIV and STI testing and PrEP knowledge and uptake. Our secondary outcomes will compare the incidence of laboratory-confirmed STI between the 2 arms. This pilot RCT will enroll a sample of 160 YMSM and their partners (a total of 320 individuals). Self-completed assessments will be conducted online, and self-collected samples for STI testing will be collected every 3 months across the intervention and control arms, with a total follow-up period of 9 months. Recruitment and eligibility screening will mirror the procedures for the prior phases.

#### Registration and Randomization

Study procedures will mirror phase II. After registering, assented participants will complete a 25-min baseline questionnaire and will then be randomly assigned in a 1:1 ratio into the intervention or control condition. Participants in the intervention condition will be mailed HIV test kits immediately after their first session and those in the control condition will be mailed HIV test kits immediately after their baseline assessments are complete.

#### Control Condition

The index participant and their partner who are randomized to the control condition will engage in only 1 telehealth session: the existing CHTC intervention delivered via video counseling. In the existing CHTC session, couples receive all elements of counseling and testing together: pretest counseling, HIV testing, discussion of HIV risks, delivery of test results, and posttest counseling. Sessions are future focused: participants are not asked to reveal recent risk behaviors/exposures. Instead, the focus is on the couple learning their serostatus together and building a prevention plan that reflects their relationship goals and serostatus. Foundational to CHTC is the couple talking about and forming a prevention plan together using effective communication skills.

Approximately 1 week before the scheduled session, a box containing 2 home HIV testing kits will be mailed to an address provided by the participants. The participants will be instructed to have the kits with them at the time of the scheduled session but not to use them before the session. During the session, the remotely located counselor will instruct the couple on how to self-test using the kits. The counselor will observe the testing and ensure they can read and interpret the results correctly, and prevention planning will be centered on the results of the HIV testing.

#### Intervention Condition

The *We Prevent* intervention will be delivered as outlined in phase II.

### Primary Outcomes

Our primary outcomes relate to the uptake of HIV prevention, conceptualized as self-reported condomless sex, HIV and STI testing, and PrEP knowledge and use. In addition, our secondary outcome will be laboratory-confirmed STIs (syphilis, gonorrhea, and chlamydia), whereby we will provide participants with kits to self-collect samples that will be mailed back and laboratory tested for STIs.

*HIV testing:* The baseline survey will include questions on lifetime HIV testing history. Follow-up surveys will assess HIV testing in the prior 3-month period and will include self-reported test results. The primary HIV testing outcome will be the proportion of YMSM tested for HIV 2 or more times, at least 3 months apart, in the 9-month follow-up period. As an additional analysis, we will also examine the proportions of participants who receive at least one HIV test.

*STIs and STI testing:* The STI testing outcome is defined as the proportion of YMSM tested for STIs at least once in the 9-month follow-up period. At baseline, we will assess lifetime STI testing history and knowledge about STIs. We will ask participants what STIs they have been tested for, the date of their most recent STI test (if known), and whether a medical provider had diagnosed them with an STI. In the follow-up assessments, participants will be mailed a box containing sample self-collection kits at each study assessment point (0, 3, 6, and 9 months). The box contains instructions on how to collect the samples and how to mail them back to the study site. The samples will be laboratory tested for syphilis, gonorrhea, and chlamydia. We will calculate the incidence of any STI in the 9-month follow-up period.

*PrEP awareness and intentions:* Surveys will assess participants’ knowledge of PrEP, willingness to use PrEP, and uptake of PrEP. PrEP awareness will be a single item measure of whether the participant has heard of PrEP [45]. PrEP willingness will be measured with an existing 8-item scale (alpha=.84) developed for YMSM to gauge likelihood of PrEP use across different conditions (eg, partner types and experiencing potential side effects) [49]. At each follow-up assessment, PrEP-eligible HIV-negative YMSM will be asked whether they have begun using PrEP, and self-reported adherence to PrEP will be assessed at each follow-up visit.

*Sexual risk behavior:* Sexual risk behavior will be assessed using the Sexual Practices Assessment Schedule [26,50] to capture the number of occasions of sex acts with different partner types, use of condoms during the past 3 months, and knowledge about partners’ HIV status and PrEP use. At-risk sex will be defined as any anal intercourse without condoms or PrEP that occurs with a person of known positive or unknown serostatus during the follow-up period.

#### Dyadic Measures

We will use the 27-item RELO-IMB scale, which was developed with items from the Health and Relationships Survey [42,43]. Information will be assessed with 5 items that gauge beliefs about HIV prevention within relationships (eg, if 2 people have sex only with each other, they really do not have to practice safer sex). Motivation will be assessed with 3 scales assessing participants’ attitudes, social norms, and intentions of using different prevention strategies for sexual risk reductions. Behavioral skills include self-efficacy to engage in preventive behaviors in different contexts and communication with partners.

#### Linkage to HIV Care

For any incident HIV-positive individuals, we will also collect the following outcomes as indicators of linkage to care, per the recent recommendations of the Institute of Medicine [51]. These are measured within 3 months of HIV diagnosis via self-report: (1) attending at least one clinical care appointment, (2) having at least one CD4 test performed, and (3) having at least one viral load test performed. Onset of antiretroviral therapy (ART) initiation, self-reported adherence to ART, and viral suppression are exploratory indicators, as we recognize that our follow-up period may not be a sufficient amount of time to see these changes.

#### Feasibility and Acceptability

In addition to the outcomes above, the pilot RCT will assess feasibility by examining (1) time to recruit 160 YMSM to the intervention and (2) rate of recruitment per 100 YMSM expressing interest in participation. Adequate feasibility will entail recruiting and enrolling at least five to six YMSM and their partners per month and ensuring at least 80% to 90% retention rate. In addition, we will examine the feasibility and acceptability of mailing home STI kits to participants. Acceptability of the intervention will be determined by analysis of data from a satisfaction survey on the intervention’s acceptability. In addition, the percentages of YMSM who do not complete either of the intervention sessions will be assessed. We will also administer a break-up survey in the event that couples break up over the course of the study. The break-up survey will assess reasons for relationship dissolution, including whether the study had an impact on their relationship.

### Phase III Data Analysis

We hypothesize that the opportunity to learn relationship skills, the experience of HIV testing with a partner, and developing a prevention plan will encourage YMSM to continue utilizing these skills throughout their relationship and in future relationships. With guidance from the iTech AC, we will analyze data at the individual level, not at the dyadic level—we expect behavioral shifts over the 9-month period among individual YMSM. Therefore, our sample for analysis will be 320 participants. We will also conduct exploratory dyadic analysis to examine how partner effects shape HIV prevention uptake among coupled YMSM.

We will examine differences between the treatment groups for the index participants using *t* tests or Wilcoxon rank sum tests for continuous variables and chi-square tests for categorical variables. We will conduct analyses of our primary and secondary HIV and STI testing behavior outcomes using regression to compare each active treatment group with the control in pairwise comparison tests. The proportion of index participants who obtain at least two tests at least 3 months apart within the follow-up period will be calculated and presented with corresponding 95% exact binomial CIs.

The ability of the intervention to increase PrEP knowledge and willingness to use PrEP over time will be examined using 2 separate outcomes. Scores at baseline and all follow-ups will be analyzed using generalized linear models (GLMs), with properly chosen (based on the distribution of dependent variable) link functions to analyze longitudinal PrEP outcome data. The GLMs will be estimated using generalized estimating equations (GEEs) with robust SE estimates, which provide an extension of regression analysis to the case of correlated or repeated observations with appropriate modeling of the covariance structure. Models will control for demographic characteristics and study arm and will explore interactions between treatment arm and individual characteristics.

The incidence of at-risk sex acts will be calculated as an incidence density, with the numerator being the number of individual at-risk sex acts and the denominator being person-years of follow time. Comparisons of the incidence of at-risk sex acts and incidence of STIs will be made by comparing incidence densities across the arms. Period incidence rates (3-monthly incidence density rates) of at-risk sex will be estimated by performing a GEE Poisson regression analysis of the 3-monthly counts, implemented using SAS PROC GENMOD. GEE models will control for demographic characteristics, baseline HIV testing history and relationship dynamics, and hypothesized mediators. GEE models will also examine interactions between relationship dynamics and sexual risk-taking. In addition, analysis will consider differences in changes in information, motivation, and behavioral skills in accordance with the RELO-IMB model.

### Cost Analysis

To inform the future development and potential scale-up of *We Prevent*, a cost analysis will be conducted for the intervention. Data on costs associated with the delivery of the intervention will be recorded over time. Capital equipment cost (eg, computers), staffing (eg, interventionists time), and facility cost (eg, rent and telephone) that are attributable to our intervention will be obtained from accounting records. No costs associated with research data collection (surveys and biomarkers) will be included. These components of cost will be summed over the 12-month study period for each participant, to generate an estimated per person cost for intervention delivery.

## Results

The *We Prevent* protocol was launched in June 2017, with phases I and II expected to be complete by mid-2019. It is expected that the pilot RCT will be launched at the end of 2019, with results finalized by mid-2021.

## Discussion

This paper describes the development of *We Prevent*, a theory-based intervention that seeks to adapt existing CHTC protocols and pair them with relationship skills counseling, both delivered through a telehealth platform, to provide the behavioral skills to reduce HIV risk in YMSM’s current and future relationships. We will draw on theory and our phase I qualitative data and phase II one-arm pilot to develop and test a dyadic intervention that will empower and enable YMSM to communicate with their partners about HIV and develop a prevention plan.

This project offers several innovations in advancing HIV prevention for YMSM in relationships. First, this intervention seeks to empower adolescent and younger MSM aged 15 to 19 years to choose goals other than 1 specific HIV prevention strategy (eg, repeat HIV testing, PrEP, or establishing sexual agreements). Existing dyadic HIV interventions, such as CHTC, have addressed sexual agreement building. However, to our knowledge, only the 2GETHER pilot intervention has incorporated relationship and HIV prevention education to produce skill building among young same-sex male couples. *We Prevent* builds on this premise and uses MI techniques to enhance motivation and allow for goal flexibility in prevention options among adolescent and younger men in their first relationships. Couples-focused interventions that build on existing brief, motivational-focused interventions may appeal to a wider audience by offering more goal choices, especially for adolescent and younger MSM in their teens. Importantly, *We Prevent* is designed to help YMSM set HIV prevention goals within their relationships, which are likely to be transferred to other relationships over time. CHTC holds promise when delivered using video-based counseling for MSM [26]; therefore, this project will build on this work to adapt the telehealth intervention for YMSM. Intervention research using mobile technology is urgently needed with YMSM communities at risk for HIV who may not have access to services (eg, rural areas). Thus, we believe that *We Prevent* has the potential to reduce HIV and other STIs among YMSM by providing young men with the motivation and skills necessary to manage their relationships throughout their lifetime.

## Abbreviations

AC: analytic core
ART: antiretroviral therapy
CAS: condomless anal sex
CDC: Centers for Disease Control and Prevention
CHTC: couples HIV testing and counseling
GEE: generalized estimating equation
GLM: generalized linear model
HIPAA: Health Insurance Portability and Accountability Act
IDI: in-depth interview
iTech: innovative technology
LGBTQ: lesbian, gay, bisexual, transgender, queer
MI: motivational interviewing
MSM: men who have sex with men
PrEP: pre-exposure prophylaxis
RELO-IMB: Relationship-Oriented Information-Motivation-Behavioral Skills
RCT: randomized controlled trial
STI: sexually transmitted infection
SRV: subject recruitment venue
TC: technology core
TEG: technical expert group
YAB: youth advisory board
YMSM: young gay, bisexual, and other men who have sex with men
